# Dynamics of Malaria Incidence in Khyber Pakhtunkhwa, Pakistan: Unveiling Rapid Growth Patterns and Forecasting Future Trends

**DOI:** 10.1007/s44197-024-00189-6

**Published:** 2024-02-14

**Authors:** Muhammad Imran Khan, Humera Qureshi, Suk Joo Bae, Adil Shah, Naveed Ahmad, Sadique Ahmad, Muhammad Asim

**Affiliations:** 1 Department of Industrial Engineering, Hanyang University, Seoul, South Korea; 2 Health Department, Peshawar, Khyber Pakhtunkhwa Pakistan; 3 EIAS: Data Science and Blockchain Laboratory, College of Computer and Information Sciences, Prince Sultan University, 11586 Riyadh, Saudi Arabia

**Keywords:** Infection, Khyber Pakhtunkhwa, KP, Malaria, Prediction, Pakistan

## Abstract

**Background:**

Malaria remains a formidable worldwide health challenge, with approximately half of the global population at high risk of catching the infection. This research study aimed to address the pressing public health issue of malaria’s escalating prevalence in Khyber Pakhtunkhwa (KP) province, Pakistan, and endeavors to estimate the trend for the future growth of the infection.

**Methods:**

The data were collected from the IDSRS of KP, covering a period of 5 years from 2018 to 2022. We proposed a hybrid model that integrated Prophet and TBATS methods, allowing us to efficiently capture the complications of the malaria data and improve forecasting accuracy. To ensure an inclusive assessment, we compared the prediction performance of the proposed hybrid model with other widely used time series models, such as ARIMA, ETS, and ANN. The models were developed through R-statistical software (version 4.2.2).

**Results:**

For the prediction of malaria incidence, the suggested hybrid model (Prophet and TBATS) surpassed commonly used time series approaches (ARIMA, ETS, and ANN). Hybrid model assessment metrics portrayed higher accuracy and reliability with lower MAE (8913.9), RMSE (3850.2), and MAPE (0.301) values. According to our forecasts, malaria infections were predicted to spread around 99,301 by December 2023.

**Conclusions:**

We found the hybrid model (Prophet and TBATS) outperformed common time series approaches for forecasting malaria. By December 2023, KP’s malaria incidence is expected to be around 99,301, making future incidence forecasts important. Policymakers will be able to use these findings to curb disease and implement efficient policies for malaria control.

## Introduction

In Khyber Pakhtunkhwa (KP), Pakistan, malaria remains a major public health problem [1], posing numerous challenges to healthcare systems and communities. Due to the high prevalence and persisting transmission rates of malaria, the region’s healthcare infrastructure and socio-economic development are under a lot of strain. For effective intervention strategies and resource allocation, you must understand the historical context and current landscape of malaria incidence in KP.

A comprehensive assessment of the current malaria situation in KP and a prediction of future growth is the goal of this study. The developed hybrid model allows us to capture the complications of malaria data and improve forecast accuracy by combining Prophet and TBATS (Trigonometric seasonality, Box–Cox transformation, ARMA errors, Trend, and Seasonal components) methods. To make sure a comprehensive evaluation, we compared the forecast performance of our hybrid model with other widely used time series models, like Auto-Regressive Integrated Moving Average (ARIMA), Exponential Smoothing State Space Model (ETS), and Artificial Neural Network (ANN) [2–5]. The aim of this extensive comparison is to find the most accurate predicting method for malaria estimation to facilitate the development of more reliable and timely disease management strategies.

The KP province has been seriously facing the burden of malaria [1], experiencing a rapid surge in infection rates in recent years. The unexpected growth of malaria cases in KP necessitates an inclusive understanding of the current trend to forecast future infection strength and establish timely and targeted intervention strategies. For disease prevention, control, and treatment, understanding the trend for future malaria progress is essential for policymakers, public health authorities, and healthcare experts to develop and implement effective strategies. By anticipating possible challenges and patterns, this research aims to contribute valuable insights to the efforts for curbing the growth of malaria infections in KP, ultimately safeguarding the health and well-being of its population.

This study delves into an inclusive evaluation of prevailing research related to malaria incidence in KP, organized into distinct themes to ensure a logical and coherent structure. First, an exploration of factors influencing malaria incidence serves as a foundational theme. Various studies have investigated multifaceted determinants contributing to the prevalence of malaria in KP, encompassing ecological factors like climate and environmental conditions, socio-economic variables, human behaviors, and the impact of vector control measures. Notably, the research by Mazhar et al. [6] and Bashir et al. [7] highlighted the correlation between climate change and malaria incidence, emphasizing the necessity of understanding environmental factors in disease transmission. Additionally, the work of Khan et al. [5] underscored the role of socio-economic disparities in malaria prevalence, shedding light on the significance of targeted interventions for vulnerable populations.

The second thematic area pertains to malaria trends and forecasting methodologies, which have been extensively explored in various studies. Anwar et al. [8] utilized ARIMA models to predict malaria outbreaks, demonstrating the applicability of time series analysis in forecasting disease patterns. Additionally, studies conducted by Parveen et al. [9] and Santosh et al. [2] underscored the potential of artificial intelligence, particularly Artificial Neural Networks (ANN), for malaria forecasting, adeptly capturing intricate data relationships. In line with these research endeavors, our study aims to significantly contribute by employing a hybrid model that integrates Prophet and TBATS methods, striving for enhanced accuracy in predicting malaria growth within KP. This approach allows for a comprehensive comparison with existing time series models such as ARIMA, ETS, and ANN. The literature review provides a succinct summary of pivotal findings while emphasizing existing methodological gaps, setting the stage for our innovative approach towards forecasting malaria dynamics.

Apparently, KP does not have much research on malaria transmission patterns and predictors. To solve this, we analyzed data from the Integrated Disease Surveillance & Response System (IDSRS), KP. By utilizing advanced prediction methods to integrate Prophet and TBATS models, this research endeavors to offer reliable forecasts regarding malaria’s future spread and pace in that region. To mitigate malaria’s adverse effect on the well-being of KPs, these forecasts provide lucid and significant insights, allowing policymakers and healthcare experts to craft specific strategies.

## Methods

### Study Area

**Fig. 1:**
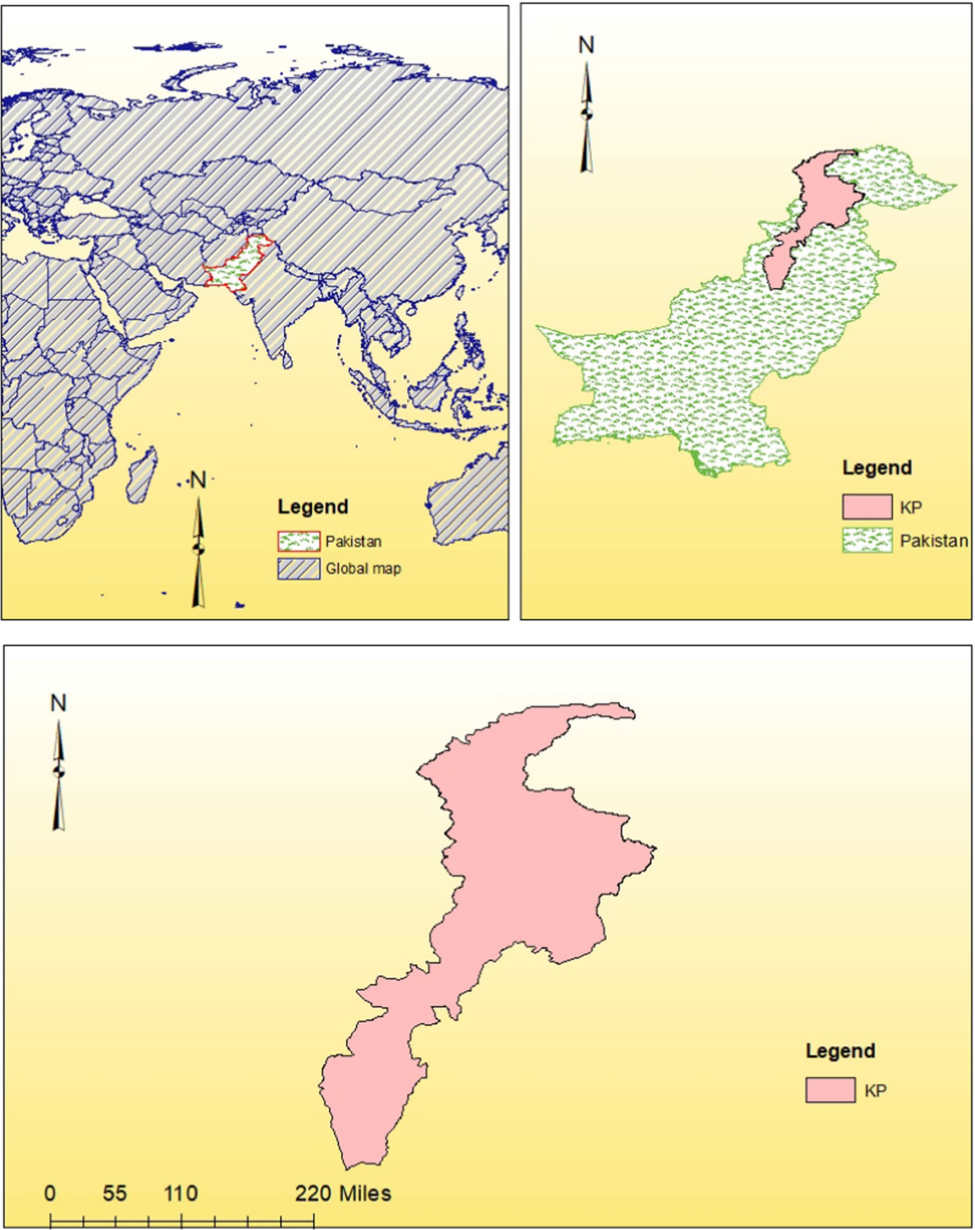
Study area map

The study area for this paper is Khyber Pakhtunkhwa (KP) province (Fig. 1), located in the northwestern region of Pakistan [5]. KP province covers a total area of about 101,741 square kilometers, the province is connected to Afghanistan to the north and west, northeast to Gilgit-Baltistan, east to Azad Jammu and Kashmir, and southeast to Punjab.

The province experiences monsoon patterns and the southwest monsoon brings rainfall from July to September. These monsoon rains are important for agricultural activities. But, they also pose the risk of floods and landslides, especially in hilly areas. The unique combination of landscapes and changing climates in the study area provides an opportunity for research across various fields such, as public health, environmental sciences, social sciences, agriculture, and more [10]. ArcGIS version 10.8 was used to design the study area map.

### Data Collection

The data were collected from the Integrated Disease Surveillance & Response System (IDSRS) of KP province, comprising a period of 5 years from 2018 to 2022. The data include several descriptive parameters linked to malaria, such as the total number of malaria tests conducted, the number of malaria confirmed cases, and the specific subtypes of malaria infections, such as *P. vivax*, *P. falciparum*, and mixed infections.

Moreover, for estimation purposes, the study explicitly focused on a subset of the data, accurately the month-wise malaria cases from January 2021 to December 2022. This subset of data permits in-depth analysis of the trends and patterns of malaria cases monthly over this specific period. The data collection processes intricate retrieval of relevant healthcare records and reports from the provincial health department.

### Hybrid Model Approach

The hybrid model employed in this study pooled the forecasting capabilities of the Prophet and TBATS models to forecast malaria cases for the year 2023 in KP province. The Prophet model is a time series forecasting model that develops a decomposable additive model with seasonality and trend components. It integrates historical patterns, offering flexibility in capturing several time series patterns. The TBATS model, on the other hand, is a specialized time series model that grips multiple seasonalities and incorporates Box–Cox transformation, ARMA errors, trends, and seasonal components.

In the hybrid model, the Prophet component incorporates a piecewise-linear trend function (or “growth term”) ($g(t)$), capturing the growth term, and seasonal patterns ($s(t)$). It also considers the effects of holidays ($h(t)$) and includes a white noise error term ($\varepsilon (t)$) to account for random fluctuations [11]. The core body of the TBATS model contains five key components. ${\text{Level}}(t)$ signifies overall average behaviors of time series; $Trend(t)$ signifies long-term direction or growth/decay of time series; $Seasonal(t)$ captures repeating patterns within a single season; the error term $\epsilon (t)$ represents random variations or noises in time series that are not accounted for by the other components [12]. Finally, for the observed value of the time series at time $t, y(t)$, the proposed hybrid model is given as:

1 $$y\left( t \right) = g\left( t \right) + s\left( t \right) + h\left( t \right) + \varepsilon \left( t \right) + {\text{Level}}\left( t \right)*{\text{Trend}}\left( t \right)*{\text{Seasonal}}\left( t \right)*\left( t \right).$$

Prior to applying the hybrid model, the raw data were preprocessed to confirm its appropriateness for analysis. This process comprised checking for missing values, handling outliers, and transforming the data if necessary. The hybrid model was trained using historical data up to a certain cutoff point. The dataset was separated into a training set and a test set, with the training set used to train the model and the test set used to assess its performance. The Prophet and TBATS models were distinctly trained on the training set to capture fundamental outlines and seasonality. The trained models were then used to make predictions on the test set.

### Forecasting

Once the hybrid model was evaluated and deemed suitable, it was useful to the whole dataset to estimate malaria cases for the year 2023 in KP province. The model applied historical patterns and seasonality captured during the training phase to produce predictions for future periods. The models were developed through R-statistical software (version 4.2.2).

## Comparison Methods

In this study, we employed an effective hybrid model integrating Prophet and TBATS methods. This innovative approach permitted us to capture the intricacies of malaria data and improve predicting accuracy. To endorse robust evaluation, we checked the forecast performance of our hybrid model with other widely used time series models, e.g., ARIMA, ETS, and ANN. By accompanying this comparison, we aimed to identify the most effective prediction method for malaria prediction, paving the way for more reliable and timely disease management strategies.

Auto-Regressive Integrated Moving Average (ARIMA) is an important time series forecasting method that captures both autoregressive and moving average components. ARIMA has an advantage in handling time series data that are not stationary. It achieves stationarity by differencing the data, which makes it suitable for predicting short to medium-term tasks and capturing linear relationships within the data. Additionally, the ability to adjust model parameters such as the order of moving terms provides flexibility, in capturing various patterns found in time series data [5, 8, 13].

2 $$Y_{t} = \beta_{1} Y_{t - 1} + \beta_{2} Y_{t - 2} + \cdots + \beta_{p} Y_{t - p} - e_{t} + \emptyset_{1} e_{t - 1} + \emptyset_{2} e_{t - 2} - \cdots \emptyset_{q} e_{t - q} ,$$

where $\beta$ and $\varnothing$ are the coefficients of the AR and MA processes, $p$ and $q$ are the past values of “${Y}_{t}$” and the error terms, respectively. The general notation of ARIMA model written as ARIMA ($p$, $d$, $q$) where “$p$” is an order of AR component, “$d$” is an order of differencing, and “$q$” is an order of MA component.

The Exponential Smoothing State Space (ETS) model is another significant approach for forecasting time series, that is simple to employ and flexible to various kinds of time series data. ETS models are supremely useful when there is a trend or seasonality in the data. Their flexibility in modeling an extensive variety of time series patterns stems from their capability to handle both additive and multiplicative components in the data. Moreover, ETS models are commonly ideal for short-term prediction tasks due to their simplicity and affluence of implementation, specifically when data patterns are realistically stable [4, 14].

3 $${\text{F}}_{t} = \alpha {\text{A}}_{{t - {1}}} + \, ({1} - \alpha ){\text{ F}}_{{t - {1}}} , {\text{t }} > \, 0$$

where *α* is the smoothing factor, and 0 < *α* < 1.

**Fig. 2:**
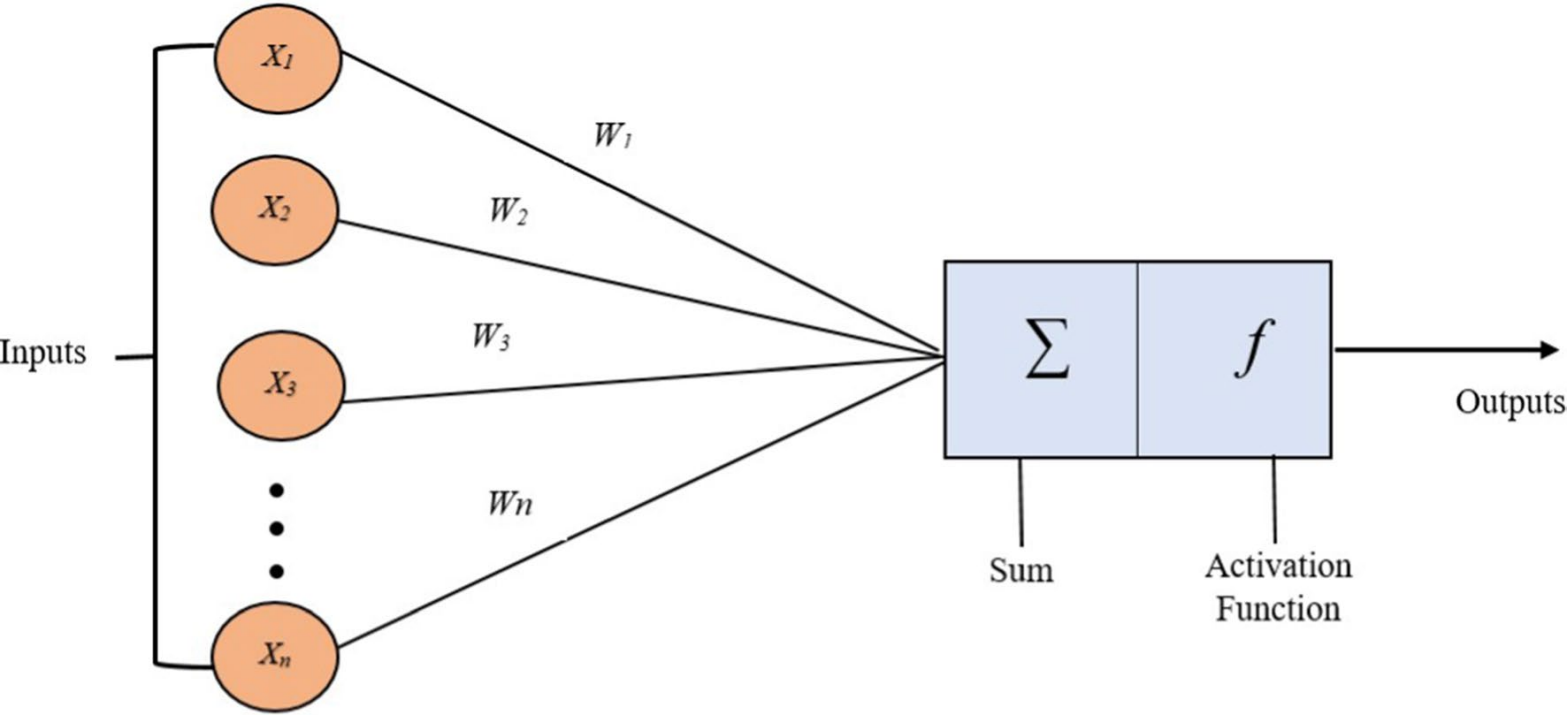
Feed forward network

Artificial Neural Networks (ANN) are advanced machine learning approaches that excel at classifying tough and non-linear relationships in time series data. Its strength is its capability to learn from historical patterns and identify complex dependencies, which makes it well-suited for forecasting tasks with diverse and large datasets. ANN models, contrasting traditional statistical approaches, do not entail robust assumptions about data distribution and can detect ultimate patterns automatically. Moreover, ANN models can handle missing data and multiple input variables, permitting them to capture interactions between various factors that influence time series (Fig. 2) [2, 15].

### Model Evaluation and Selection

To evaluate the accuracy of the models, several assessment metrics were employed. These included Mean Absolute Error (MAE), Root Mean Squared Error (RMSE), and Mean Absolute Percentage Error (MAPE). These metrics provided insights into the model’s fit, accuracy, and predictive performance.

## Results

**Fig. 3:**
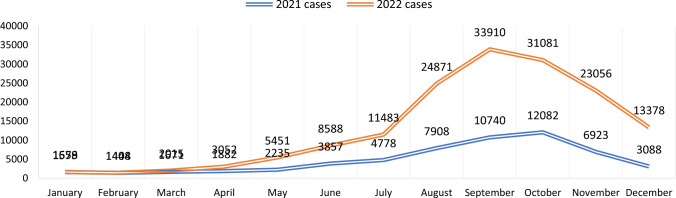
Month-wise malaria cases comparison for the years 2021 and 2022

Over the past 5 years, figures for malaria prevalence (Table 1) portray that a notable increase occurred in 2022. In that year, a total of 206,557 malaria suspects underwent screening, and 159,912 individuals were confirmed positive for malaria. Historically, *P. vivax* has been the major predominant species, contributing to approximately 90.6% of the disease burden, while *P. falciparum* accounted for the remaining 9.4%. The cumulative Annual Parasite Incidence (API) for all districts in KP in 2022 was reported as 4.56, with an Annual Blood Examination Rate (ABER) of 5.9% and a Test Positivity Rate (TPR) of 8%. When comparing the malaria data with the previous year 2021, the number of cases has shown a threefold increase throughout the province (Fig. 3).

**Table 1:** Malaria year-wise data of settled districts of Khyber Pakhtunkhwa

Year	Malaria tests	Malaria confirmed	PV	PF	Mix	TPR %	API	ABER %
2018	1,049,214	113,642	110,157	3077	408	10.8	3.6	3.3
2019	1,067,705	81,460	79,420	1929	111	7.6	2.5	3.3
2020	1,298,856	54,176	52,582	1488	106	4.2	1.6	3.9
2021	1,506,671	58,226	54,754	3362	110	4	1.71	4.4
2022	2,065,957	159,912	144,911	13,732	1269	8	4.56	5.9

*PV Plasmodium vivax*; *PF* *Plasmodium falciparum*; *Mix* *Plasmodium vivax + Plasmodium falciparum*; *TPR* test positivity rate; *API* annual parasite incidence; *ABER* annual blood examination rate

The results of our study demonstrate that the hybrid model, combining Prophet and TBATS techniques, outperformed other commonly used time series models, such as ARIMA, ETS, and ANN, in forecasting malaria prevalence. The hybrid model’s superior performance was evident in its ability to capture intricate patterns of malaria occurrence and provide more accurate forecasts.

**Fig. 4:**
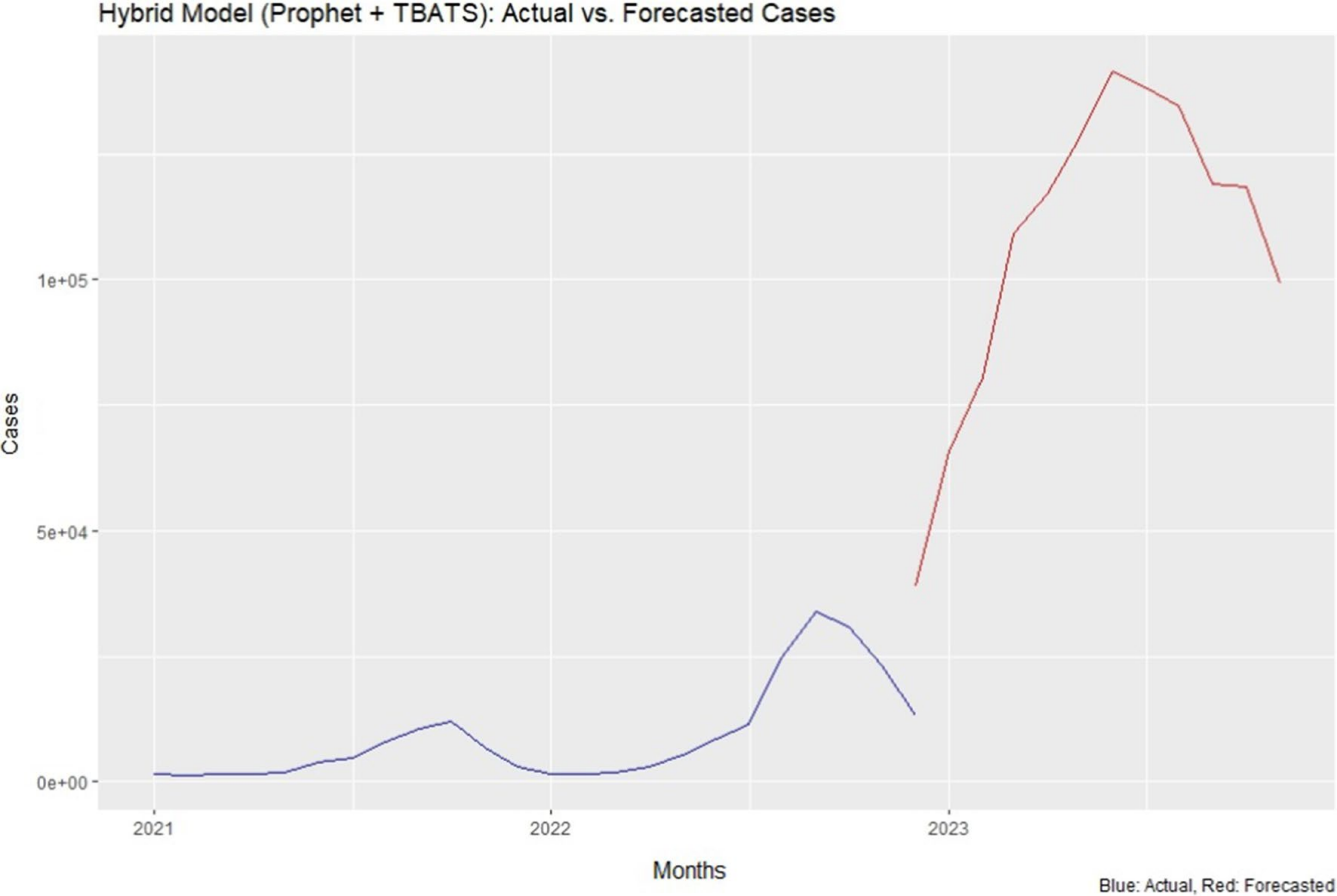
Hybrid model (Prophet and TBATS) more effectively explains the estimation of malaria cases trend with an MAE (8913.9) and RMSE (3850.2) values. It implies that its predictions had smaller deviations from the actual data. The predicted line shows a significant increase for the upcoming month (up to the end of December 2023)

According to our predictions, malaria cases prevalence is projected to reach approximately 99,301 by the end of December 2023. Figure 4 visually illustrates the predicted increase in confirmed cases over time, showcasing the hybrid model’s ability to closely match the actual data trends.

In terms of evaluation metrics, the hybrid model achieved lower values for MAE (Mean Absolute Error), RMSE (Root Mean Squared Error), and MAPE (Mean Absolute Percentage Error) compared to other comparative models (Table 2). These metrics are indicative of the accuracy and reliability of the forecasting models. The hybrid model’s lower MAE (8913.9) and RMSE (3850.2) values imply that its predictions had smaller deviations from the actual data, while the lower MAPE (0.301) value indicates that its percentage errors were significantly reduced.

**Table 2:** Models evaluation metrics

Models	Metrics		
	MAE	RMSE	MAPE
Hybrid	8913.9	3850.2	0.301
ARIMA	13,208.8	15,791.6	0.641
ETS	7139.35	7994.6	0.434
ANN	19,564.6	21,129.3	0.712

*MAE* Mean absolute error; *RMSE* root mean squared error; *MAPE* mean absolute percentage error

The encouraging results highlight the effectiveness of the hybrid model (Prophet and TBATS) for malaria prediction, showcasing its potential to support public health authorities and policymakers in implementing timely and targeted disease management strategies. By improving forecasting accuracy, the hybrid model can play a pivotal role in enhancing preparedness and response efforts to combat malaria outbreaks, ultimately contributing to better health outcomes for affected populations.

## Discussion

Malaria remains a global health challenge, resulting in significant mortality each year [16]. Our study aimed to estimate malaria incidence in KP using a hybrid model that combines the Prophet and TBATS models, generating insights into disease dynamics and prediction for the region. Our findings indicated that the hybrid model outperforms traditional time series models (ARIMA, ETS, and ANN) in comparison criteria, presenting its capacity to capture subtle data patterns for more accurate forecast. Using this hybrid technique, we forecast a malaria incidence of around 99,301 by the end of December 2023. Prophet is well-known for handling seasonal and trend components, while TBATS is proficient in handling cyclic patterns and nonlinearities in the data. By combining these techniques, the hybrid model was able to take various factors that affect malaria prevalence, resulting in more precise and reliable forecasts into account [11, 12, 17, 18].

Our study findings compared to existing studies, the prevalence of malaria in KP province indicated a threefold increase across the region (Table 1). Approximately 90.6% responsible *P. vivax* of the disease burden, while *P. falciparum* responsible 9.4%. This is consistent with prior research indicating *P. vivax* dominance in malaria cases [19]. While our hybrid model supported detailed estimates, current literature often lacked thorough, localized predictions using advanced predictive methods.

The prevalence of malaria in KP during 2022 was influenced by various risk factors stemming from climatic changes, such as floods and heavy rainfall [20, 21]. Additionally, the unpredictable transmission patterns, poor socio-economic conditions, and declining health infrastructure further compounded the disease burden. The low immune status of the population, resource constraints, and limited access to preventive and curative services also played significant roles. Moreover, mass population movements within the country and across international borders contributed to the spread of the disease [19]. The key factors contributing to the increase in malaria cases in KP during 2022 are as follows. Devastating floods struck Pakistan, affecting over 33 million people and leading to the declaration of 81 districts as calamity-hit areas. As per the LLIN distribution strategy, universal coverage for the targeted rural population was not been achieved in any target district. The intake of an incomplete dose of Primaquine fails to completely clear the gametes from the patient’s body, therefore, malaria transmission continues despite its treatment. Community education plays an important role in filling the gap in treatment compliance and in adopting preventive practices. There is a lake of effective community education intervention in the ongoing malaria control program.

While our study emphasizes the benefit of the hybrid model in forecasting malaria incidence compared to other generally used time series methods, it is essential to admit specific limitations in our methodology. First, the precision of the estimates relies strongly on the completeness and quality of the input data. Any missing or incorrectness data in the historical data might affect the functioning of the intended hybrid approach. Second, though the hybrid model integrates Prophet and TBATS methods successfully, the selection of these specific approaches may not be ideal for all locations or scenarios. There might be alternative models or variations that could generate better results in distinct contexts. Furthermore, as with any prediction approach, improbabilities are fundamental and unexpected factors like variations in mediation strategies or climate patterns could change the precision of future estimates. Thus, while our study furnishes significant perceptions into malaria incidence trends, cautious interpretation of the forecasts must be guaranteed, and complementary methods and further studies are encouraged to enhance forecasting capabilities in this domain.

## Conclusion

The superior performance of the hybrid model is attributed to its ability to efficiently capture intricate data patterns, leading to more accurate and reliable forecasts. With our projections, we estimate that malaria incidence will be expected to reach about 99,301 cases by the end of December 2023. The combination of Prophet’s expertise in handling seasonal and trend components with TBATS' proficiency in managing cyclic patterns and nonlinearities permitted the hybrid model to take several factors influencing malaria prevalence into account, resulting in more accurate and reliable predictions. These findings emphasize the significance of using the hybrid model for anticipating and handling future trends in malaria prevalence in the KP province of Pakistan.

## Abbreviations

ABER: Annual blood examination rate
ANN: Artificial neural network
API: Annual parasite incidence
ARIMA: Auto-regressive integrated moving average
ETS: Exponential smoothing state space model
IDSRS: Integrated disease surveillance & response system
IRS: Indoor residual spraying
KP: Khyber Pakhtunkhwa
LLIN: Long lasting insecticide-treated nets
MAE: Mean absolute error
MAPE: Mean absolute percentage error
*P. falciparum*: *Plasmodium falciparum*
*P. vivax*: *Plasmodium vivax*
RMSE: Root mean squared error
TBATS: Trigonometric seasonality, box–cox transformation, ARMA errors, trend, and seasonal components
TPR: Test positivity rate
WHO: World health organization
